# Severe lower limb infection by *Kerstersia gyiorum*: clinical and genomic insights into an underestimated pathogen

**DOI:** 10.3389/fmed.2025.1639069

**Published:** 2025-09-12

**Authors:** Jiayuan Qin, Guangmin Tang, Yu Feng, Xiaochao Hu, Yanbin Liu, Xiaoju Lv, Fang He

**Affiliations:** ^1^Center of Infectious Diseases, West China Hospital, Sichuan University, Chengdu, China; ^2^Division of Infectious Diseases, State Key Laboratory of Biotherapy, Sichuan University, Chengdu, China; ^3^Center for Pathogen Research, West China Hospital, Sichuan University, Chengdu, China; ^4^Luzhou People's Hospital, Luzhou, China

**Keywords:** *Kerstersia gyiorum*, skin and soft tissue infection, whole-genome sequence, clinical characteristics, genomic features

## Abstract

Since *Kerstersia gyiorum* was first described and named in 2003, reports of human infections caused by this organism have gradually increased. Here, we present a detailed report of a severe case of lower right limb infection caused by *K. gyiorum* that was characterized by rapid disease progression and multidrug resistance. We also present the complete genome sequence of the isolate, WCHKG1. A systematic analysis of the clinical features of our case patient and previous *K. gyiorum*-infected patients revealed that the most common site of infection was the lungs (48%), and that the organism showed the lowest sensitivity to commonly used quinolones among the major antibiotic classes. Clinical infections caused by *K. gyiorum* may be underestimated, thus the use of quinolones in treating such infections should be avoided. Genomic and phylogenetic analyses of *K. gyiorum* identified conservation of antibiotic efflux pump systems and virulence factors, which may play critical roles in its antibiotic resistance and pathogenicity. Furthermore, evidence of clonal transmission in animals suggests a need for vigilance regarding potential clonal spread in clinical settings. Our study contributes to the current understanding of *K. gyiorum* and offers useful insights to support its clinical management and infection control.

## 1 Introduction

*Kerstersia gyiorum*, a Gram-negative coccobacillus belonging to the genus *Kerstersia* within the family *Alcaligenaceae*, was first described and named by Coenye et al. (1). The species name *gyiorum*, meaning “from the limbs,” was chosen because the organism was initially primarily isolated from lower-extremity wounds (1). However, *K. gyiorum* has since been detected in a variety of environments, including the intestines of brown-throated sloths (2), the blowholes of Yangtze finless porpoises (3), and boar semen (4), indicating a broad ecological distribution. For nearly a decade after its initial identification, there were no reports of *K. gyiorum* causing human clinical infections. Almuzara et al. (5) reported a case of cholesteatomatous chronic otitis media caused by *K. gyiorum*, followed an increasing number of studies documenting infections involving the ear (6), lower limbs (6), lungs (7), urinary tract (8), appendix (9), and bloodstream (10) in a human. Importantly, *K. gyiorum* is difficult to distinguish from other microorganisms using conventional methods, including biochemical tests and automated identification systems. This is primarily due to its biochemical phenotype being similar to other pathogens (such as *Alcaligenes faecalis*) (11), as well as its delayed inclusion in automated biochemical identification system databases (e.g., Vitek2) (5), leading to past misidentification or oversight in clinical laboratories (10, 12, 13). Consequently, its clinical significance may have been overlooked or underestimated. Previous studies have reported that *K. gyiorum* may be misidentified as *Alcaligenes faecalis* (5), *Aeromonas salmonicida* (14), or *Lautropia mirabilis* (10), which can delay accurate species identification and impact the timely implementation of appropriate treatment strategies. With the advancement of techniques such as matrix-assisted laser desorption/ionization time-of-flight mass spectrometry (MALDI–TOF MS) and 16S rRNA gene sequencing, *K. gyiorum* can now be identified more accurately in clinical laboratories (15, 16). Furthermore, the maturation and widespread use of next-generation sequencing (NGS) have facilitated the acquisition of complete genome sequences for *K. gyiorum* isolates (2, 17, 18). However, a systematic analysis of the clinical and genomic characteristics of *K. gyiorum* is urgently needed to improve our understanding of this organism and establish a reliable foundation for infection prevention and treatment. In this study, we report a case of severe lower limb infection caused by *K. gyiorum* that was successfully treated. Additionally, we conducted a comprehensive analysis of the clinical and genomic features of *K. gyiorum* to help fill the current knowledge gap regarding this emerging pathogen.

## 2 Materials and methods

### 2.1 Bacterial isolation, culture, and identification

Colombian blood agar and MacConkey agar plates (Pangtong Medical Devices, Chongqing, China) were inoculated with wound exudate specimens and incubated at 37°C for 24 h in a 5% CO_₂_ incubator. The species of the isolated strains were identified using MALDI–TOF MS. The *K. gyiorum* strain WCHKG1 was further purified, and its colony morphology was observed. Species identification was confirmed via 16S rRNA gene sequencing, as previously described (11).

### 2.2 Morphological characterization

Gram staining was performed using a commercial staining kit (Bomei Biotechnology, Hefei, China). Bacterial morphology and the Gram reaction were observed under a light microscope (Olympus, Tokyo, Japan) using oil immersion. Negative staining of WCHKG1 was performed using 2.0% (w/v) uranyl acetate, and its fine morphological features were observed under a transmission electron microscope (Hitachi, Japan) at an accelerating voltage of 80 kV.

### 2.3 Antimicrobial susceptibility testing

The minimum inhibitory concentrations (MICs) of antibiotics against WCHKG1 were determined using the broth microdilution method, in accordance with the Clinical and Laboratory Standards Institute (CLSI) guidelines (19). *Escherichia coli* ATCC 25922 was used as the quality control strain, and all tests were conducted in triplicate. Except for tigecycline, whose breakpoint was defined by the European Committee on Antimicrobial Susceptibility Testing (20), breakpoints for all other antibiotics were interpreted in accordance with CLSI criteria (19). Antimicrobial susceptibility testing for additional isolates was performed using the Vitek2-Compact analysis system (bioMérieux, France) and the Kirby-Bauer disk diffusion method.

### 2.4 Whole-genome sequencing

Genomic DNA of strain WCHKG1 was extracted using the QIAamp DNA Mini Kit (Qiagen, Hilden, Germany) and sequenced using the HiSeq X Ten platform (Illumina, San Diego, CA, United States), in accordance with the manufacturers’ protocols. For long-read sequencing, DNA from the same batch was prepared using the Rapid Sequencing Kit V14 (Oxford Nanopore Technologies, Oxford, United Kingdom), and sequencing was conducted on the MinION platform using an R10.4.1 flow cell. Paired-end 150-bp reads were trimmed to remove adapters using Trimmomatic v0.39 (21) and assembled into contigs using SPAdes v3.15.3 (22). For complete genome assembly, reads shorter than 1,000 bp or with an average quality score < Q8 were filtered out using NanoFilt v2.8.0 (23). To obtain the complete genome, a hybrid assembly of short and long reads was performed using Unicycler v0.4.3 (24). Default parameters were used for all bioinformatic tools unless otherwise specified.

### 2.5 Patients and clinical data

Clinical data collected from the patient reported in this study included comprehensive demographic information, background conditions, comorbidities, infection site, specimen type, diagnostic methods, identified pathogens, antimicrobial susceptibility results, antibiotic treatments, and clinical outcomes. In addition, we conducted literature searches of the PubMed, Web of Science, Embase, Ovid MEDLINE, Cochrane, bioRxiv, and medRxiv databases to identify all studies reporting human infections caused by *K. gyiorum* that had been published up to March 15, 2025. The search terms used were “*Kerstersia gyiorum*” and “*K. gyiorum*.” We also reviewed the reference lists of the retrieved articles. Studies published in both English and non-English languages were included, and Google Translate was used for translations of studies in languages other than English or Chinese. Relevant patient characteristics and infection-related information were extracted from all eligible reports and analyzed.

### 2.6 Genomic analysis

Whole-genome sequences of *K. gyiorum* available in the GenBank database as of March 15, 2025, were retrieved and analyzed alongside our genome sequence of WCHKG1. Quality control of the assembled genomes was performed using QUAST v5.3.0 (25) and CheckM2 v1.1.0 (26). Species identification was based on average nucleotide identity (ANI) analysis using fastANI v1.34 (27). Plasmid replicons, antimicrobial resistance genes, and virulence factors were identified using ABRicate^1^ in conjunction with the PlasmidFinder (28), ResFinder (29), and VFDB (30) databases. Given the limited characterization of plasmids, resistance genes, and virulence factors in *K. gyiorum*, we applied relaxed thresholds of 60% for both percent identity and subject coverage. Single-nucleotide polymorphisms (SNPs) in the *K. gyiorum* genomes were identified using Snippy v4.6.0.^2^

### 2.7 Phylogenetic analysis

Core genes across the *K. gyiorum* genome sequences were identified and aligned using PIRATE v1.0.5.^3^ Homologous regions were identified and removed using Gubbins v3.4 (31). A maximum-likelihood phylogenetic tree was then constructed based on the genomic sequences with homologous recombination regions removed, using the GTRGAMMA model with 1,000 bootstrap replicates. The tree was visualized using iTOL.^4^

## 3 Results

### 3.1 Case overview

**Figure 1:**
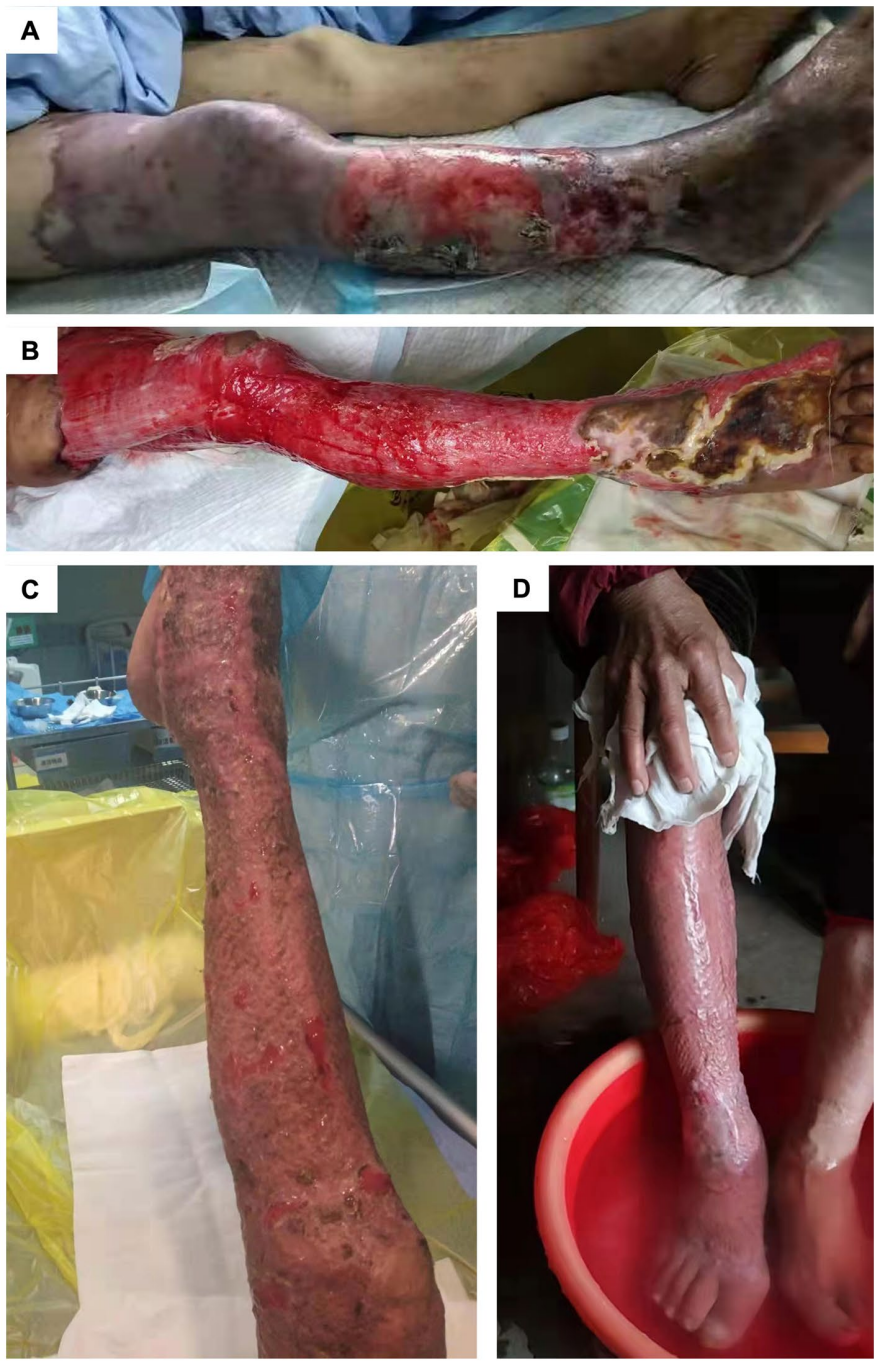
Severe lower limb infection caused by *Kerstersia gyiorum*. **(A)** Condition of the patient’s lower limb upon hospital admission. **(B)** Progression of the infection at 1 week post admission. **(C)** Postoperative appearance following skin grafting. **(D)** Condition of the lower limb at 6 months post discharge.

A 69-year-old female patient was admitted to the hospital with a 2-year history of swelling and pain, a 1-month history of ulceration in the right lower limb, and intermittent low-grade fever for 1 week. Upon admission, her highest recorded body temperature was 38.0 °C, without accompanying chills or rigors. She was a farmer by occupation, with no history of smoking or alcohol consumption. At admission, her comorbidities included varicose veins of the right lower limb, renal insufficiency, hyperuricemia, malnutrition, and anemia. On physical examination, the patient’s vital signs were as follows: respiratory rate 21 breaths/min, heart rate 82 beats/min, blood pressure 122/68 mmHg, and oxygen saturation (SpO_₂_) 98% without supplemental oxygen. Examination of the right lower limb revealed swelling and tenderness, extensive erythema, localized skin ulceration with purulent discharge, partial epidermal desquamation, and a foul odor (Figure 1A). By 1 week post admission, the skin necrosis on the right lower limb had progressed rapidly, with peeling of the necrotic tissue and exposure of the underlying muscle. Wound debridement was performed to remove necrotic epidermis and purulent exudate from the affected leg (Figure 1B). Laboratory tests revealed elevated inflammatory markers: white blood cells (14.96 × 10^9^/L), neutrophils (11.73 × 10^9^/L), procalcitonin (0.45 ng/mL), C-reactive protein (98.90 mg/L), and interleukin-6 (117 pg./mL). Additionally, liver and kidney function tests showed normal total serum bilirubin, alanine aminotransferase, and aspartate aminotransferase levels, but elevated serum creatinine (99.00 μmol/L) and reduced estimated glomerular filtration rate (eGFR) at 48.64 mL/min/1.73 m^2^. Abdominal venous Doppler ultrasound showed no abnormalities in the inferior vena cava, bilateral common iliac veins, or external iliac veins. The patient was initially treated empirically with intravenous broad-spectrum antibiotic imipenem (1.0 g every 12 h) for 1 week prior to confirmation of the causative organism.

Wound exudate from the patient’s right leg was collected and used to inoculate Colombian blood agar and MacConkey agar plates. Four distinct colony morphologies were observed. MALDI-TOF MS identified the bacteria from these colonies as *K. gyiorum*, *Proteus mirabilis*, *Pseudomonas aeruginosa*, and *Providencia stuartii*. In three subsequent cultures of wound secretions collected on non-consecutive days, only *K. gyiorum* was isolated, suggesting that *K. gyiorum* was the primary and persistent pathogen responsible for the lower limb wound infection. *K. gyiorum* was further purified and designated as strain WCHKG1. After a 24-h culture on Colombian blood agar and MacConkey agar, WCHKG1 formed flat, gray-white, slightly dry colonies with an irregular surface and relatively regular edges (Supplementary Figure S1). Light microscopic examination and staining revealed that the strain was Gram-negative, displaying single, paired, or bead-like chain arrangements (Supplementary Figure S2). Under transmission electron microscopy, WCHKG1 cells measured approximately 1 μm in length and 0.8 μm in width; no flagella were observed, but surface folds were present (Supplementary Figure S3). A 1,395-bp 16S rRNA gene sequence was obtained for WCHKG1. BLAST analysis against the NCBI core nucleotide database identified *K. gyiorum* as the closest match, with 99.93% identity to the type strain LMG 5906 (GenBank accession number NR_025669.1).

Antimicrobial susceptibility testing of *K. gyiorum* WCHKG1 showed the following: susceptible (S) to amikacin, ampicillin, aztreonam, cefazolin, cefepime, cefotaxime, ceftazidime, ceftriaxone, gentamicin, imipenem, meropenem, minocycline, piperacillin, piperacillin–tazobactam, tigecycline, tobramycin, and trimethoprim–sulfamethoxazole; intermediate (I) to chloramphenicol and colistin; and resistant (R) to cefuroxime, ciprofloxacin, levofloxacin, and tetracycline (Table 1). *P. mirabilis*, *P. aeruginosa*, and *P. stuartii* were susceptible to amikacin, aztreonam, meropenem, piperacillin–tazobactam, and ceftazidime. *P. mirabilis* and *P. stuartii* were resistant to trimethoprim–sulfamethoxazole, ciprofloxacin, moxifloxacin, and levofloxacin. Based on the susceptibility profile, the patient’s antibiotic regimen was adjusted to intravenous infusion of piperacillin–tazobactam (4.5 g every 8 h) for 4 weeks. She received anti-infective treatment, along with meticulous wound care and nutritional support. As a result, the right lower limb wound infection was brought under control, purulent discharge was reduced, and the patient’s body temperature returned to normal. The patient subsequently underwent skin grafting on the granulating wound of the right lower limb (Figure 1C). Following intensive treatment, inflammatory markers normalized, the skin graft survived, and she was discharged with an improved condition. Six months after discharge, the patient returned to normal daily life (Figure 1D). A 3-year follow-up showed that the patient did not experience recurrent infection, although a residual limp remained (Supplementary Figure S4).

**Table 1:** Minimum inhibitory concentration (MIC) values of *Kerstersia gyiorum* WCHKG1.

Antibiotic	MIC (μg/mL)	Susceptibility
Amikacin	2	S
Ampicillin	0.5	S
Aztreonam	2	S
Cefazolin	8	S
Cefepime	2	S
Cefotaxime	0.5	S
Ceftazidime	1	S
Ceftriaxone	≤0.125	S
Cefuroxime	32	R
Chloramphenicol	16	I
Ciprofloxacin	64	R
Colistin	0.5	I
Gentamicin	1	S
Imipenem	0.25	S
Levofloxacin	8	R
Meropenem	≤0.03	S
Minocycline	4	S
Piperacillin	0.5	S
Piperacillin-tazobactam	0.5/4	S
Tetracycline	16	R
Tigecycline	0.5	S
Tobramycin	0.5	S
Trimethoprim-sulfamethoxazole	≤0.125/2.375	S

S, susceptible; R, resistant; I, intermediate.

### 3.2 Clinical characteristics of patients with *Kerstersia gyiorum* infection

A total of 158 articles were initially retrieved from the literature. After removal of duplicates, 43 articles were screened by title and abstract. Studies were included in the subsequent analysis if they clearly identified *K. gyiorum* as the causative pathogen and reported infections in human patients. Ultimately, 22 articles met the eligibility criteria. These studies collectively reported a total of 39 cases of *K. gyiorum* infection (5–14, 16, 17, 32–41) (Supplementary Table S1), which were analyzed alongside our case to summarize the clinical characteristics. Among these 40 patients, the median age was 68 years (range: 13–95 years) and 68% (*n* = 27) were male. The most common country of origin was China (52.5%, *n* = 21), followed by Turkey (15%, *n* = 6) and the United States (12.5%, *n* = 5) (Supplementary Table S1). The majority of patients (53%, *n* = 21) had comorbidities associated with *K. gyiorum* infection (Supplementary Table S1).

Analysis of infection sources revealed that the most common site of infection was the lungs (48%, *n* = 19), followed by the ears (28%, *n* = 11), and lower limbs (18%, *n* = 7) (Supplementary Table S1). The most frequently collected specimen types were wound secretions (45%) and sputum (45%) (Supplementary Table S1). Thirty-seven (93%) of the patients were diagnosed with *K. gyiorum* infection using MALDI-TOF MS, while three (7%) were identified via 16S rRNA gene sequencing. In 12 studies involving 14 patients, the VITEK system was reportedly unable to accurately identify *K. gyiorum* (Supplementary Table S1). More than half (68%, *n* = 27) of the cases involved polymicrobial infections, which included our case. The most commonly co-isolated pathogen was *P. aeruginosa*, found in 37% (10/27) of the mixed-infection cases (Supplementary Table S1). Treatment and clinical outcomes were documented in 22 patients, of whom 16 (73%) received monotherapy with antibiotics as initial treatment, and six (27%) received combination antibiotic therapy. Quinolones were the most common initial antibiotics (50%). The total duration of antibiotic therapy ranged from 4 to 70 days. Most of these patients (91%, 20/22) recovered, with one patient experiencing residual sequelae (Supplementary Table S1).

### 3.3 Antibiotic susceptibility of *Kerstersia gyiorum*

A total of 35 *K. gyiorum* isolates from 18 previous studies underwent antimicrobial susceptibility testing (5–14, 16, 18, 34, 36–40). These results were integrated with the susceptibility profile of WCHKG1 (Table 2). *K. gyiorum* demonstrated high susceptibility to the following antibiotics: imipenem (100%, 29/29), meropenem (100%, 30/30), ceftazidime (94%, 31/33), amikacin (94%, 29/31), piperacillin–tazobactam (92%, 22/24), cefepime (90%, 18/20), tobramycin (86%, 19/22), gentamicin (82%, 18/22), and aztreonam (83%, 19/23). In contrast, resistance was frequently observed to ciprofloxacin (71%, 25/35), levofloxacin (48%, 12/25), and trimethoprim–sulfamethoxazole (21%, 6/29) (Table 2).

**Table 2:** Antibiotic susceptibility profiles of all *Kerstersia gyiorum* isolates.

Numbers	AMK	AMP	ATM	CFZ	CPM	CTX	CAZ	CRO	CXM	CHL	CIP	COL	GEN	IPM	LVX	MEM	MIN	PIP	PIT	TET	TGC	TOB	TMP-SMX	References
1	S	S	S	S	S	S	S	S	R	I	R	I	S	S	R	S	S	S	S	R	S	S	S	This study
2	S		S		S		S				R		S	S	S	S			S			S	S	Egyir et al. (18)
3					R	I	R	S			R								R				S	Bostwick et al. (10)
4	S		R		S		S				R	S		S	I	S			S				R	Uysal et al. (13)
5	S		S				S	S			S	R	S	S		S		S	S				S	Baran et al. (11)
6	S		S		S	S	S				R		S	S	S	S		S	S			S	S	Kim et al. (12)
7	S	S			S	S	S						S	S	S	S		S	S					Ogawa et al. (8)
8	S	S	S	S	S		S	S			S		S	S	S				S			S	S	Lan et al. (34)
9	S				S		S	S			S		S	S	S		S						S	Almuzara et al. (5)
10	I	R		R	I	R	S		R		R		R	S	S	S			S			R	R	AlSunbul et al. (36)
11	S		S		S		S				R			S	R	S			S			S	S	Sun et al. (14)
12	S		S		S		S				R			S	R	S			S			S	S	Sun et al. (14)
13	S				S						I		S						S			S	S	Deutscher et al. (7)
14	S						S	S			R		S	S							S			Borsa et al. (37)
15	S						R				R		R	S		S			S					Özcan et al. (16)
16	S				S	S	S	S			S		R										R	Özcan et al. (16)
17	S				S	S	S	S			R		R			S							R	Özcan et al. (16)
18						S	S				S		S	S		S		S						Mwalutende et al. (38)
19						S	S				S		S	S		S		S						Mwalutende et al. (38)
20					S						R		S			S			S				S	Pence et al. (6)
21					S						R		S			S			S				S	Pence et al. (6)
22	S		S				S				R			S	I	S			S	S	S	S	S	Zhang et al. (39)
23	R		S				S				R			S	R	S			S	R	I	R	R	Zhang et al. (39)
24	S		R				S				R			S	R	S			R	S	R	R	R	Zhang et al. (39)
25	S		S				S				R			S	R	S				S	S	S	S	Zhang et al. (39)
26	S		S				S				R			S	R	S			S			S		Zhang et al. (39)
27	S		S				S				I			S	S	S				S	S	S	S	Zhang et al. (39)
28	S		S				S				R			S	R	S						S		Zhang et al. (39)
29	S		S				S				R			S	R	S				S	S	S	S	Zhang et al. (39)
30	S		S				S				R			S	R	S				S	S	S	S	Zhang et al. (39)
31	S		R				S				R			S	R	S				S	S	S	S	Zhang et al. (39)
32	S		S		S		S	S			I		S	S	S	S		S	S			S	S	Jiang et al. (40)
33	S		S		S		S	S			R		S	S	I	S		S	S			S	S	Jiang et al. (40)
34	S		S		S		S	S			R		S	S	R	S		S	S			S	S	Jiang et al. (40)
35	S		S		S		S	S			R		S	S	S	S		S	S			S	S	Jiang et al. (40)
36	S		R				S	S			S		S		S	S			S	I			S	Xiao et al. (9)

AMK, Amikacin; AMP, Ampicillin; ATM, Aztreonam; CFZ, Cefazolin; CPM, Cefepime; CTX, Cefotaxime; CAZ, Ceftazidime; CRO, Ceftriaxone; CXM, Cefuroxime; CHL, Chloramphenicol; CIP, Ciprofloxacin; COL, Colistin; GEN, Gentamicin; IPM, Imipenem; LVX, Levofloxacin; MEM, Meropenem; MIN, Minocycline; PIP, Piperacillin; PIT, Piperacillin-tazobactam; TET, Tetracycline; TGC, Tigecycline; TOB, Tobramycin; TMP-SMX, Trimethoprim-sulfamethoxazole. Blank cells indicate that susceptibility data for the corresponding antibiotic were not available for the isolate.

### 3.4 Genomic characteristics of *Kerstersia gyiorum*

The complete genome of *K. gyiorum* strain WCHKG1 contains 3,884,372 bp, with a sequencing coverage of 845 × and a GC content of 62.41%. This strain shares 98.85% ANI with the type strain *K. gyiorum* DSM 16618^T^ (NCBI assembly accession no. GCA_004216755.1), exceeding the commonly accepted species demarcation threshold of 95–96% (42), thereby confirming its species identity. The complete genome sequence and Sequence Read Archive (SRA) data have been submitted to the GenBank database.

**Figure 2:**
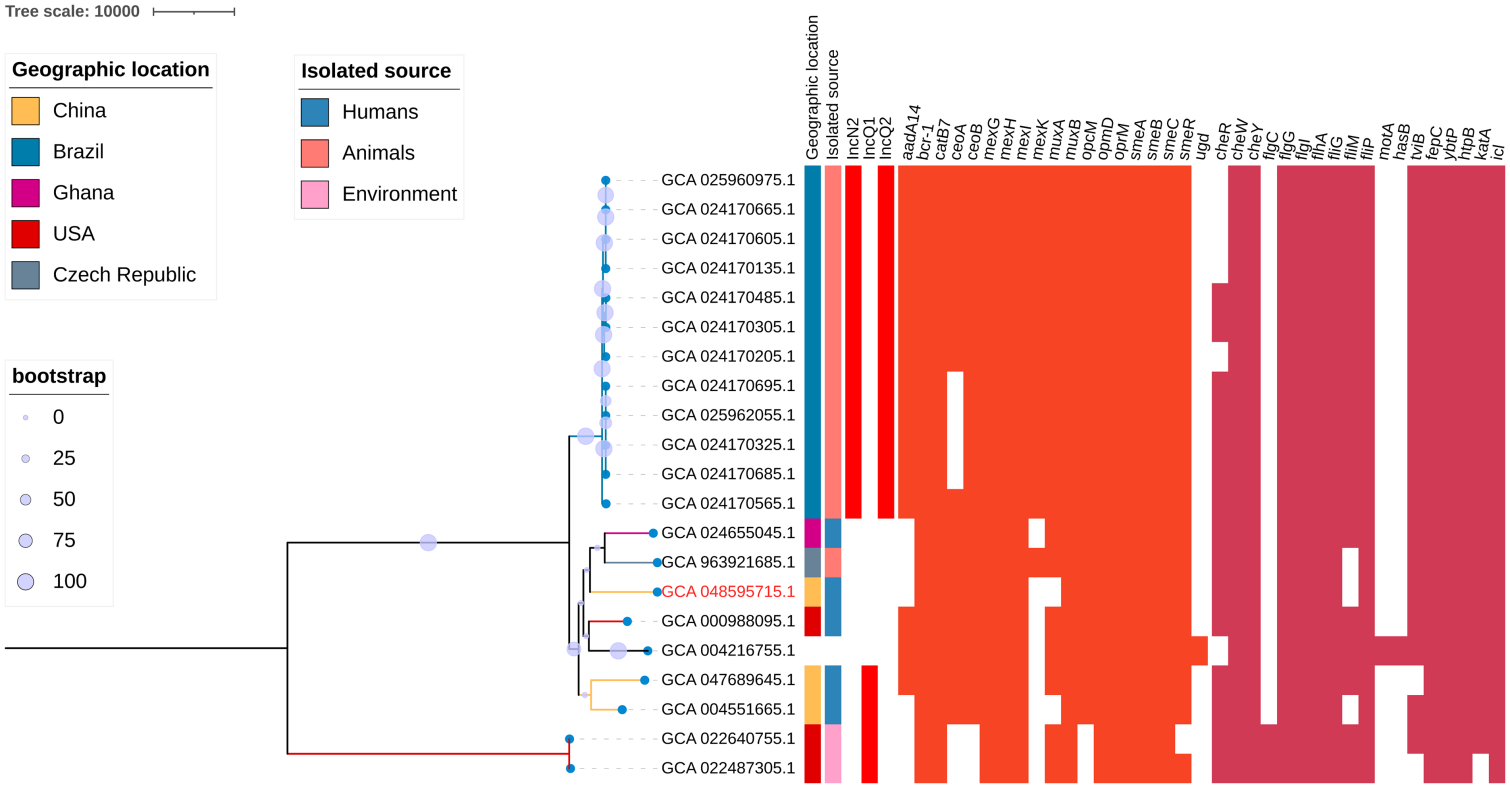
Genomic and phylogenetic characteristics of *Kerstersia gyiorum* strains. A maximum-likelihood phylogenetic tree was constructed based on genomic sequences from 21 *K. gyiorum* strains, with homologous recombination regions excluded. The tree scale represents point mutations. Bootstrap values are indicated by circles of varying sizes, with larger circles corresponding to higher bootstrap values. The strain sequenced in this study (GCA_048595715.1) is highlighted in red. Colored blocks and white spaces indicate the presence or absence of specific genetic elements, respectively.

We retrieved 22 *K. gyiorum* whole-genome sequences from the GenBank database.^5^ Among them, GCA_004216755.1 (strain DSM 16618), GCA_008801725.1 (strain CCUG 47000), and GCA_965138795.1 (strain CIP108214) represent the genome of the *K. gyiorum* type strain. Given the superior assembly quality of GCA_004216755.1 (strain DSM 16618), which has fewer contigs, it was selected for subsequent analyses. Ultimately, 20 *K. gyiorum* genomes were included in the final analysis. These genomes, obtained between 2014 and 2024, originated from five countries, with Brazil contributing the most (60%, 12/20). Thirteen of the 20 genomes were isolated from animal feces, five from humans (including four clinical isolates), and two from environmental sources (Supplementary Table S2; Figure 2). The genome sequence of WCHKG1 was analyzed in combination with these publicly available genomes.

All 21 genomes exhibited high completeness (> 93%) and low contamination (< 2.5%). Compared with the type strain DSM 16618^T^, the ANI values ranged from 97.93 to 100.00%, confirming that all of the genomes belong to the *K. gyiorum* species. Genome sizes ranged from 3.57 to 3.98 Mbp (average: 3.83 Mbp) and GC content varied between 62.36 and 62.69% (Supplementary Table S2). Plasmid replicons IncN2, IncQ1, and IncQ2 were detected among these genomes. IncN2 and IncQ2 were present in most (92%, 12/13) of the animal-derived isolates, but were absent in human- and environment-derived isolates. Conversely, IncQ1 was detected in 33% (2/6) of the human-derived isolates and 100% (2/2) of the environment-derived isolates, but was not found in animal-derived isolates (Supplementary Table S2; Figure 2).

Using the ResFinder database, 19 genes associated with antimicrobial resistance were identified (Supplementary Table S2; Figure 2). These included the following: genes encoding efflux pumps, such as CeoAB-OpcM, which are associated with reduced susceptibility to aminoglycosides and fluoroquinolones (43); MexGHI-OpmD, affecting resistance to fluoroquinolones and tetracyclines (44); MuxABC-OpmB, impacting resistance to aztreonam, macrolides, tetracycline, and novobiocin (45); and the smeABC complex, which influences fluoroquinolone susceptibility (46). These resistance determinants were relatively well conserved across the *K. gyiorum* genomes (Supplementary Table S2; Figure 2).

Using the VFDB database, 18 putative virulence factors were identified (Supplementary Table S2; Figure 2). These included genes related to capsule formation, flagella, lipopolysaccharide (LPS), and siderophore systems, such as enterobactin and yersiniabactin. Notably, genes involved in flagellar biosynthesis (*cheW, cheY, flgG, flgI, flhA, fliG, fliP*), enterobactin-related transport (*fepC*), and yersiniabactin biosynthesis (*ybtP*) were highly conserved and detected in all of the analyzed *K. gyiorum* genomes (Supplementary Table S2; Figure 2).

### 3.5 Phylogenetic characteristics

A maximum-likelihood phylogenetic tree was constructed based on genomic sequences from 21 *K. gyiorum* strains, with homologous recombination regions excluded (Figure 2). Phylogenetic analysis revealed a host-associated clustering pattern, with genomes from humans, animals, and environmental sources forming relatively distinct clades (Figure 2). Next, we analyzed the SNPs between isolates. Among *K. gyiorum* genomes derived from humans (minimum pairwise SNPs > 1,800) and environmental sources (minimum pairwise SNPs > 300), no evidence of clonal clustering was observed (Figure 2; Table 3). By contrast, three clonal clusters were identified among the animal-derived *K. gyiorum* genomes, each characterized by < 25 SNPs between strains within the cluster and by > 500 SNPs separating them from strains outside the cluster (Figure 2; Table 3).

**Table 3:** Number of single-nucleotide polymorphisms (SNPs) between *Kerstersia gyiorum* isolates.

No. SNPs	GCA_025960975.1	GCA_024170665.1	GCA_024170605.1	GCA_024170135.1	GCA_024170485.1	GCA_024170305.1	GCA_024170205.1	GCA_024170695.1	GCA_025962055.1	GCA_024170325.1	GCA_024170685.1	GCA_024170565.1	GCA_024655045.1	GCA_963921685.1	GCA_048595715.1	GCA_000988095.1	GCA_004216755.1	GCA_047689645.1	GCA_004551665.1	GCA_022640755.1	GCA_022487305.1
GCA_025960975.1	0*	6*	10*	22*	946	947	936	1,200	1,204	1,201	1,193	1,250	22,460	23,220	22,786	19,235	24,181	22,622	19,235	44,463	46,731
GCA_024170665.1	6*	0*	8*	23*	948	949	941	1,202	1,205	1,203	1,196	1,252	22,479	23,246	22,803	19,234	24,158	22,641	19,245	44,465	46,736
GCA_024170605.1	10*	8*	0*	23*	948	947	940	1,200	1,206	1,204	1,194	1,252	22,485	23,226	22,801	19,233	24,210	22,625	19,252	44,482	46,749
GCA_024170135.1	22*	23*	23*	0*	939	940	935	1,196	1,199	1,200	1,191	1,243	22,454	23,214	22,797	19,227	24,130	22,626	19,237	44,450	46,721
GCA_024170485.1	946	948	948	939	0*	5*	527	1,173	1,176	1,179	1,175	1,226	22,461	23,223	22,836	19,239	24,176	22,656	19,244	44,505	46,781
GCA_024170305.1	947	949	947	940	5*	0*	527	1,174	1,175	1,177	1,175	1,224	22,452	23,218	22,828	19,233	24,175	22,652	19,248	44,501	46,778
GCA_024170205.1	936	941	940	935	527	527	0	1,152	1,163	1,153	1,154	1,220	22,492	23,247	22,793	19,252	24,192	22,639	19,239	44,472	46,747
GCA_024170695.1	1,200	1,202	1,200	1,196	1,173	1,174	1,152	0*	9*	14*	6*	1,207	22,491	23,261	22,880	19,269	24,255	22,691	19,258	44,427	46,779
GCA_025962055.1	1,204	1,205	1,206	1,199	1,176	1,175	1,163	9*	0*	19*	13*	1,214	22,509	23,276	22,892	19,305	24,319	22,698	19,289	44,446	46,806
GCA_024170325.1	1,201	1,203	1,204	1,200	1,179	1,177	1,153	14*	19*	0*	12*	1,209	22,522	23,283	22,869	19,297	24,312	22,714	19,311	44,484	46,836
GCA_024170685.1	1,193	1,196	1,194	1,191	1,175	1,175	1,154	6*	13*	12*	0*	1,205	22,493	23,266	22,879	19,286	24,278	22,688	19,278	44,441	46,775
GCA_024170565.1	1,250	1,252	1,252	1,243	1,226	1,224	1,220	1,207	1,214	1,209	1,205	0	22,615	23,393	22,796	19,383	24,199	22,859	19,380	44,442	46,760
GCA_024655045.1	22,460	22,479	22,485	22,454	22,461	22,452	22,492	22,491	22,509	22,522	22,493	22,615	0	23,344	24,948	22,260	27,499	25,854	22,869	47,518	49,990
GCA_963921685.1	23,220	23,246	23,226	23,214	23,223	23,218	23,247	23,261	23,276	23,283	23,266	23,393	23,344	0	24,641	23,148	26,899	26,077	24,183	47,796	50,444
GCA_048595715.1	22,786	22,803	22,801	22,797	22,836	22,828	22,793	22,880	22,892	22,869	22,879	22,796	24,948	24,641	0	22,806	25,665	25,268	22,528	47,067	49,865
GCA_000988095.1	19,235	19,234	19,233	19,227	19,239	19,233	19,252	19,269	19,305	19,297	19,286	19,383	22,260	23,148	22,806	0	26,387	23,424	18,321	44,788	47,145
GCA_004216755.1	24,181	24,158	24,210	24,130	24,176	24,175	24,192	24,255	24,319	24,312	24,278	24,199	27,499	26,899	25,665	26,387	0	27,561	24,638	49,136	51,679
GCA_047689645.1	22,622	22,641	22,625	22,626	22,656	22,652	22,639	22,691	22,698	22,714	22,688	22,859	25,854	26,077	25,268	23,424	27,561	0	20,754	47,125	49,700
GCA_004551665.1	19,235	19,245	19,252	19,237	19,244	19,248	19,239	19,258	19,289	19,311	19,278	19,380	22,869	24,183	22,528	18,321	24,638	20,754	0	45,356	48,200
GCA_022640755.1	44,463	44,465	44,482	44,450	44,505	44,501	44,472	44,427	44,446	44,484	44,441	44,442	47,518	47,796	47,067	44,788	49,136	47,125	45,356	0	341
GCA_022487305.1	46,731	46,736	46,749	46,721	46,781	46,778	46,747	46,779	46,806	46,836	46,775	46,760	49,990	50,444	49,865	47,145	51,679	49,700	48,200	341	0

*Isolates belonging to the same cluster. Cells are highlighted in pink for visibility.

## 4 Discussion

Here, we have presented a case of severe right lower limb infection caused by *K. gyiorum*, distinguishable from typical presentations of *Vibrio vulnificus*-associated necrotizing fasciitis (47) by the patient’s full-thickness skin necrosis with intact underlying muscle tissue. The atypical pattern of tissue damage may indicate a unique pathogenic mechanism, warranting further investigation. Although previous studies reported chronic lower limb infections due to *K. gyiorum* (6, 11, 32), to our knowledge, this is the most severe acute manifestation to be documented.

Strain WCHKG1, isolated from the wound exudate, was identified as *K. gyiorum* using MALDI-TOF MS and confirmed by 16S rRNA gene sequencing, consistent with previously reported diagnostic approaches (12, 13, 34). WCHKG1 exhibited resistance to multiple antibiotics, including cefuroxime, ciprofloxacin, levofloxacin, and tetracycline. Notably, earlier studies (39) also reported *K. gyiorum* strains resistant to ciprofloxacin, levofloxacin, and tetracycline, highlighting the emerging concern of antibiotic resistance in this species. Our patient was treated with a combination of antimicrobial therapy and skin grafting, and was followed for 3 years. Although she returned to her normal daily life, a persistent limp remained. This is the first reported case of *K. gyiorum* infection resulting in a long-term sequela, underscoring the potential severity of such infections and the importance of timely and aggressive management.

Our analysis of 40 *K. gyiorum* cases revealed that more than half of the patients (53%) had comorbidities and 68% had polymicrobial infections—findings consistent with previous reports (14, 36). While earlier reports suggested that *K. gyiorum* primarily causes infections of the limbs and ears (1, 14), we found that the lungs were the most common site (48%), followed by the ears (28%) and lower limbs (18%). This shift may reflect the results of two recent studies from China (39, 40), as well as the increasing use of MALDI-TOF MS, which has improved diagnostic sensitivity (48). The VITEK system has been shown to misidentify *K. gyiorum* (10, 12, 13), whereas MALDI-TOF MS offers more accurate identification (13, 33, 39), reducing the risk of misdiagnosis or underdiagnosis. In most reported cases, quinolones were used as the first-line treatment. However, our analysis of susceptibility test results indicated that *K. gyiorum* strains exhibit the lowest susceptibility to quinolones among major antibiotic classes. Thus, quinolones may not be the optimal empirical choice for treating *K. gyiorum* infections.

The plasmid replicons identified among 21 *K. gyiorum* whole-genome sequences revealed differences between animal- and human-derived strains. This observation has been previously reported (2), but was not explored in depth here because of the draft quality of most genomes, which limits the accuracy of plasmid structure analysis.

Analysis of antibiotic resistance genes in *K. gyiorum* revealed the presence of multiple conserved efflux pump systems, including CeoAB-OpcM, MexGHI-OpmD, MuxABC-OpmB, and smeABC. These systems are known to reduce susceptibility to fluoroquinolones and tetracycline, providing a genomic basis for the observed resistance patterns.

Virulence factor analysis revealed that several virulence-associated genes are highly conserved in *K. gyiorum*, including those involved in flagellar biosynthesis (*cheW, cheY, flgG, flgI, flhA, fliG, fliP*), the enterobactin siderophore system (*fepC*), and the yersiniabactin siderophore system (*ybtP*). These findings are consistent with previous studies that broadly identified flagellar and iron acquisition-related genes in *K. gyiorum* genomes (2, 35). Flagella are known to play a key role in bacterial pathogenesis (49), and both enterobactin and yersiniabactin are essential for bacterial iron uptake and virulence (50, 51). However, transmission electron microscopy of *K. gyiorum* strain WCHKG1 showed that the bacterium lacked visible flagella. Similarly, another species within the *Kerstersia* genus, *K. similis*, also exhibits aflagellar morphology (52). The functional role of these conserved flagellar biosynthesis genes in *K. gyiorum* pathogenicity remains unclear, warranting further investigation.

Phylogenetic analysis revealed three clonal clusters (SNPs < 25) among animal-derived *K. gyiorum* isolates, suggesting clonal transmission in animals. Although no clonal clustering (SNPs > 1,800) was observed among human-derived isolates, the potential for clonal spread in clinical settings cannot be ruled out and should be monitored closely.

## 5 Conclusion

This case of acute, severe right lower limb infection caused by an isolate of *K. gyiorum* was characterized by rapid progression and multidrug resistance, providing valuable clinical insights into the treatment of *K. gyiorum* infections. Our clinical and genomic analyses indicated that *K. gyiorum* can infect multiple anatomical sites, and that quinolones may not be the most suitable first-line treatment. The *K. gyiorum* genome exhibits conservation of antibiotic efflux pump systems and virulence factors, which may play important roles in its antibiotic resistance and pathogenicity. Additionally, we observed evidence of clonal transmission among animal-derived isolates, highlighting the potential for similar clonal spread in clinical settings and underscoring the need for increased vigilance. These findings contribute to addressing current knowledge gaps regarding this emerging pathogen, supporting future efforts in infection prevention and management. However, our findings are limited by the relatively small number of reported *K. gyiorum* infections and available genome sequences. Further research with larger sample sizes is needed to validate these observations.

## Data Availability

The complete genome sequence of *K. gyiorum* WCHKG1 has been deposited in GenBank (BioProject accession no. PRJNA1157788), BioSample (accession no. SAMN43522527), SRA (accession no. SRR32731179), and GenBank (nucleotide accession no. CP169556). Additional raw data used in this study are available from the corresponding author upon reasonable request.

## Footnotes

^1^ https://github.com/tseemann/abricate

^2^ https://github.com/tseemann/snippy

^3^ https://github.com/SionBayliss/PIRATE

^4^ https://itol.embl.de/

^5^ https://www.ncbi.nlm.nih.gov/datasets/genome/?taxon=206506
