# Supplementary material for: Severe lower limb infection by Kerstersia gyiorum: clinical and genomic insights into an underestimated pathogen
Source: Front Med (Lausanne). 2025 Sep 12;12:1639069. doi: 10.3389/fmed.2025.1639069 (PMC12465120; doi:10.3389/fmed.2025.1639069)
Supplement: Supplementary file 1 [file Supplementary_file_1.docx]

**Supplementary Materials**


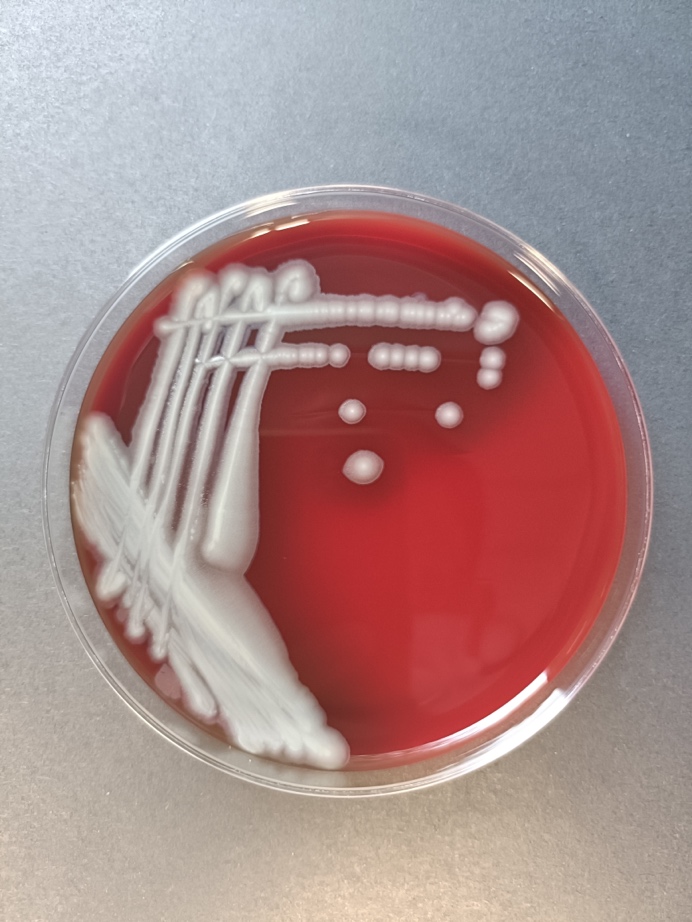
 **
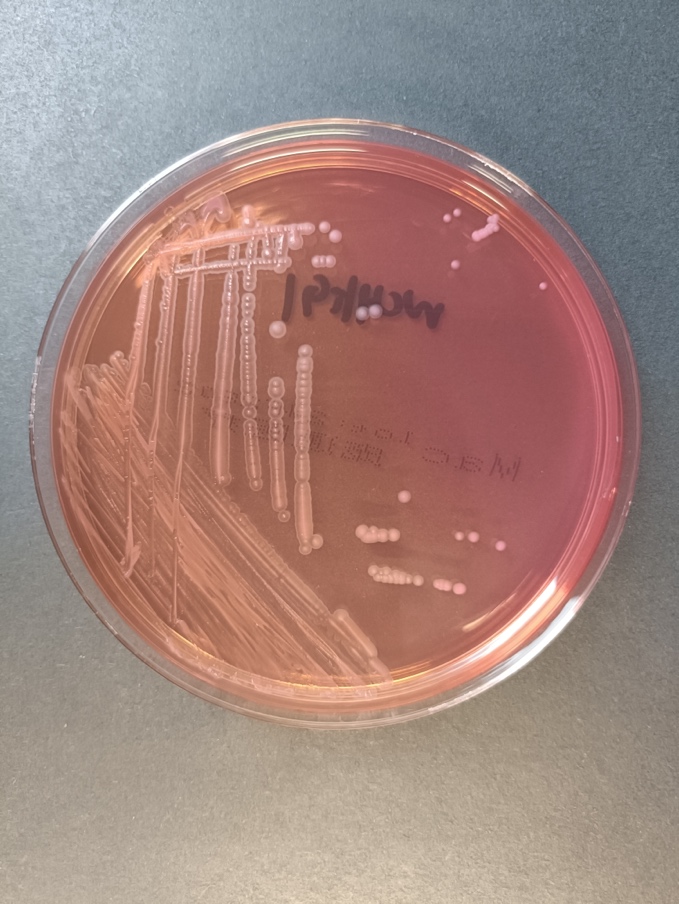
**

**B**

**A**

**Figure S1.** Colony morphology of *K. gyiorum* on culture media.

*A:* Colombian blood agar plate; *B:* MacConkey agar plate.


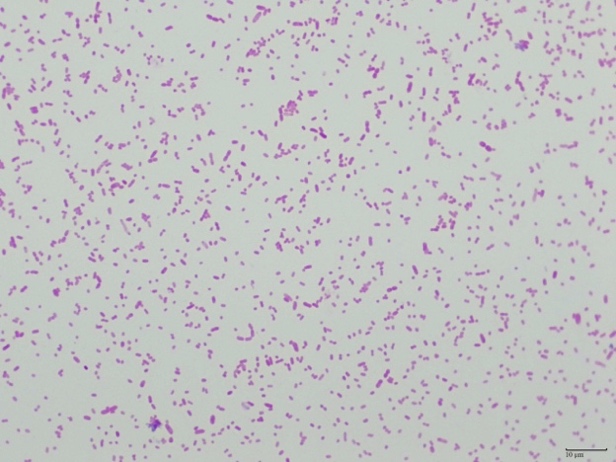


**Figure S2.** Gram staining morphology of *K. gyiorum* under microscope.

Note: Oil immersion, magnification: 1000x.


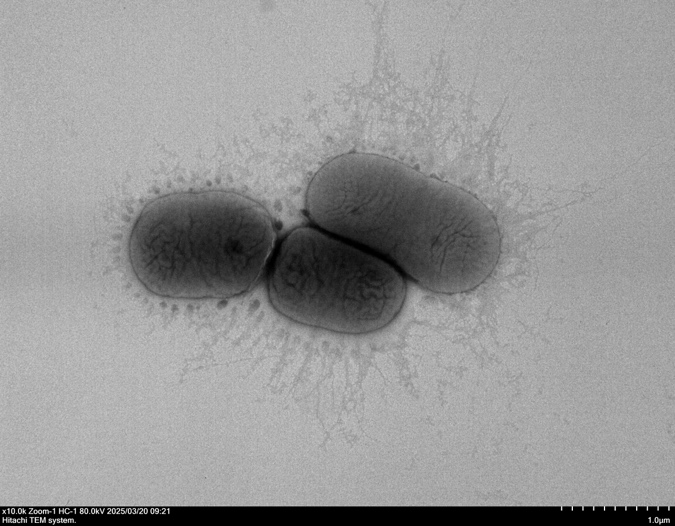


**Figure S3.** The cell morphology of *K. gyiorum* under transmission electron microscopy.

The Scale was 1 µm.

**Figure S4.** Timeline of clinical events in a case of severe right lower limb infection caused by *K. gyiorum.*

**Supplementary Table Legends**

**Table S1.** Clinical characteristics of infectious cases caused by *K. gyiorum*.

*Notes:* # indicates treatment before drug susceptibility testing; * indicates treatment after drug susceptibility testing.

**Table S2.** Genomic features of *K. gyiorum* strains.
